# HIV/TB Co-Infection in Mainland China: A Meta-Analysis

**DOI:** 10.1371/journal.pone.0010736

**Published:** 2010-05-20

**Authors:** Lei Gao, Feng Zhou, XiangWei Li, Qi Jin

**Affiliations:** State Key Laboratory for Molecular Virology and Genetic Engineering, Institute of Pathogen Biology, Chinese Academy of Medical Sciences, Beijing, China; University of Stellenbosch, South Africa

## Abstract

**Background:**

TB and HIV co-epidemic is a major public health problem in many parts of the world, particularly in developing counties. We aimed to summarize the prevalence of TB and HIV co-infection in mainland China, using meta-analysis based on systematic review of published articles.

**Methods:**

We systematically reviewed published studies, from the MEDLINE and Chinese BioMedical Literature Databases, on the prevalence of HIV infection among TB patients and on the prevalence of TB among HIV/AIDS population until 15 April 2010, and quantitatively summarized the estimates using meta-analysis.

**Results:**

In total, 29 studies were included in this review, with consistently homogeneous results. TB patients, for whom the summary prevalence of HIV infection was 0.9% (0.6%–1.4%) in mainland China, were found to be a potential target population for HIV screening. The prevalence of TB among HIV/AIDS population was 7.2% (4.2%–12.3%), but this was much higher when the analyses were restricted to AIDS patients (22.8%). Significantly higher prevalence was observed for males and hospital-based studies.

**Conclusions:**

Our analyses indicated that the prevalence of HIV/TB co-infection in China deserves special attention, screening of TB among HIV/AIDS populations should be attached more importance, which would be much more helpful for treatment of both diseases.

## Introduction

Mycobecterium tuberculosis (TB) and human immune deficiency virus (HIV) infections are two major public health problems in many parts of the world, particularly in many developing counties [1], [2]. TB is the most common opportunistic disease and cause of the death for those infected with HIV [3]. Similarly, HIV infection is one of the most important risk factors associated with an increased risk of latent TB infection progressing to active TB disease [4], [5]. An estimated 1.37 million HIV positive TB patients were diagnosed globally in 2007, and around 80% of them live in sub-Saharan Africa [6]. At least one-third of the 33.2 million people living with HIV worldwide are infected with TB and they are 20–30 times more likely to develop TB than those without HIV [6], [7]. The challenge of the TB and HIV co-epidemic has been recognized by World Health Organization, and collaborative TB/HIV activities were launched in 2004 to manage the TB and HIV co-infection [8].

The prevalence of HIV and TB within mainland China is high and the convergence of these two infectious diseases is increasingly and significantly endangering human health [9], [10]. By 1998, HIV had reached all 31 provinces and was in a phase of exponential growth. According to the estimates by the Ministry of Health and the Joint United Nations Programme on HIV/AIDS (UNAIDS), China has 560,000 to 920,000 people infected by HIV virus by 2009 (http://english.peopledaily.com.cn/90001/90782/90880/6822651.html). The 4th National Tuberculosis Epidemiological Sampling Survey, performed in 2000, indicated that the prevalence of TB infection was 44.5% and the prevalence of pulmonary TB was 367/10,000 in mainland China [11]. Multiple epidemiological studies have addressed TB and HIV co-infection in recent years [12]. However, to our knowledge, the data have not been systematically evaluated and reported in English. The objective of this study was to summarize the prevalence of TB and HIV co-infection in mainland China using a meta-analysis of published articles.

## Materials and Methods

### Literature search

Studies addressing the TB and HIV co-infection were identified by searching for articles in the MEDLINE database and Chinese BioMedical Literature Database until 15 April 2010 [13]. Various combinations of the terms “tuberculosis”, “HIV”, “AIDS”, “co-infection” and “China” were used to screen for potentially relevant studies. Additional studies were also identified using cross-referencing.

### Inclusion and exclusion criteria

Cross-sectional or cohort studies addressing the prevalence of HIV infection among TB patients or the prevalence of TB among HIV/AIDS population were included. If the study was reported in duplicate, the article published earlier was included. In the included studies, screening of HIV infection was performed by the routine ELISA test and all ELISA positives were confirmed by Western Blot. A diagnosis of TB was made based on the combined evaluation of clinical, radiological, histopathological and laboratory features of the patients in accordance with the protocol established by the National Tuberculosis Prevention and Control Program (2008): sample smears/cultures positive, or sample smears/cultures negative but meet all three of the following clinical criteria (1. symptoms consistent with TB; 2. chest X-ray suggestive of TB; 3. a positive anti-TB Rx response). Review articles, and studies in languages other than English or Chinese, with <50 participants, from the regions of China other than mainland (i.e. TaiWan, HongKong and Macao), where the diagnosis of TB was based on serology methods only, focusing on incidence rates, and addressing specific high risk populations (e.g. studies assessing HIV prevalence among TB patients who were injecting drug users), were excluded.

### Data extraction and Statistical analysis

For all included studies, we extracted the following data from original publications: first author and year of publication; study site, study base (population-based or hospital-based) and subject enrollment time; sample size and age of the participants; prevalence of HIV infection and potential route of infection; diagnosis criteria of TB and methods of differential identification of Mycobacteria in clinical samples. In population-based studies, studied cases were collected from a target population (e.g. from communities or regional hospitals); in hospital-based studies, cases were enrolled from specific hospitals and could not cover any target population. In this review, events of HIV infection and TB were extracted from the studies, if available, and included in tabular presentation. For some studies, events had to be calculated from the reported data.

Meta-analyses on the prevalence of HIV infection among TB patients and the prevalence of TB among HIV/AIDS population were carried out using the Comprehensive Meta-Analysis program (V2.0, Biostat, Englewood, NJ, USA) [14]. Stratified analyses were performed according to study base (population based or hospital based), gender of participants (male or female) and studied target population (i.e. AIDS or HIV/AIDS). Chi square test was used to assess the differences between the subgroups. Random effects models were used, taking into account the possibility of heterogeneity between studies, which was tested with the Q test (P<0.10 was considered indicative of statistically significant heterogeneity) and the I^2^ statistic (values of 25%, 50% and 75% are considered to represent low, medium and high heterogeneity respectively). Begg rank correlation and Egger weighted regression methods were used to statistically assess publication bias (p<0.05 was considered indicative of statistically significant publication bias).

## Results

**Figure 1:**
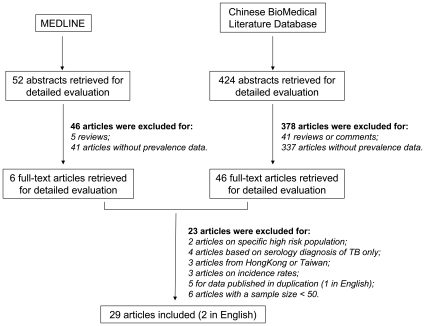
Flow diagram of study identification.

As shown in Figure 1, a total of 476 articles published in Chinese or English were identified. After excluding 46 review papers and 378 original articles without prevalence data based on abstract evaluation, 52 studies addressing TB and HIV co-infection were selected, and the full-text versions were retrieved. Of these, 23 were excluded based on the exclusion criteria (see List S1). Finally, 29 studies were included in this review [15]–[43].

**Figure 2:**
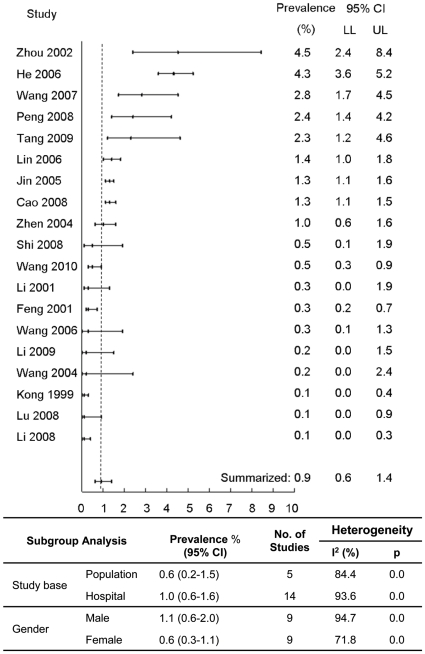
Meta-analysis of HIV infection among TB patients.

Nineteen studies, from 8 different regions or provinces, addressing HIV prevalence among TB patients are presented in Table S1 and Table S2 [16]–[34]. Fourteen of them were hospital-based and 5 were population based. Only 8 studies provided potential route of HIV infection for the studied subjects. Sample size of the studies ranged from 200 to 9887. A total of 38,101 subjects were included in the meta-analyses. Figure 2 shows the summarized estimate of HIV prevalence among TB patients in mainland China (0.9%; 95% confident interval (CI): 0.6%–1.4%; range 0.1%–4.5%). No evident publication bias was observed (p = 0.57 for Begg rank correlation analysis; p = 0.04 for Egger weighted regression analysis). The studies showed good homogeneity (Q test p<0.01; I^2^ = 92.21). Stratified analyses showed lower overall prevalence estimates from population-based studies and for females (p<0.01).

**Figure 3:**
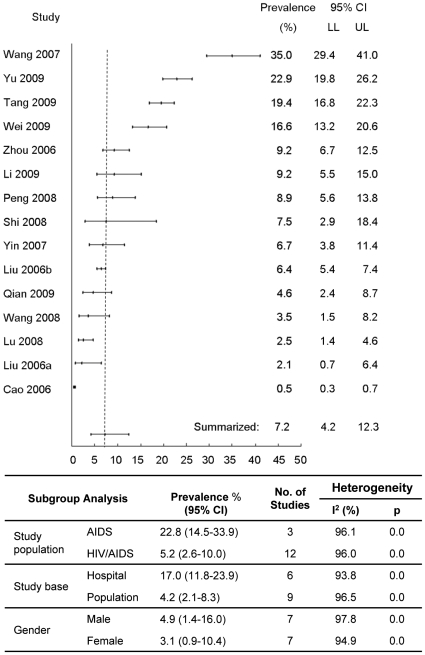
Meta-analysis of TB prevalence among HIV/AIDS population.

As shown in Table S3 and Table S4, 15 studies on the prevalence of TB among HIV/AIDS population were included and 8 of them were population-based [15], [18], [22]–[24], [35]–[43]. There are only 3 studies addressing AIDS patients and all the others addressing both HIV infections and AIDS patients. A total of 12,127 subjects were included in the meta-analyses. Figure 3 shows the summarized estimate (7.2%; 95% CI: 4.2%–12.3%; range 0.5%–35.0%). No evident publication bias was observed (p = 0.17 for Begg rank correlation analysis; p = 0.16 for Egger weighted regression analysis). No heterogeneity was found between studies (Q test p<0.01; I^2^ = 97.92). A much higher summarized prevalence was observed for studies addressing AIDS as compared to those addressing both HIV infections and AIDS patients (22.8% vs 5.2%, p<0.01). Lower prevalence was found from population-based studies and for females (p<0.01).

## Discussion

This review addressed the status of TB and HIV co-infection in mainland China. Twenty four studies were identified and summarized by meta-analysis. Our results suggest a high prevalence of TB among HIV/AIDS cases, especially among AIDS patients. Screening of TB among HIV/AIDS populations should be attached more importance in China which would be much helpful for treatment of both diseases.

To our knowledge, this is the first study to systematically review the status of TB and HIV co-infection in mainland China. TB and HIV co-infection is well recognized as a major public health problem in woldwide and many investigations have been performed and published from western countries [44]–[46]. The United Nations Joint Programme on HIV/AIDS (UNAIDS) 2009 report estimated that 33.4 million people are living with HIV in worldwide, and one third of them are co-infected with TB [47]. HIV infection increases susceptibility to TB and is the most potent factor in transferring latent or recently acquired TB infection to active clinical diseases [4], [5], [48]. In addition, TB in high HIV prevalence populations is a leading cause of morbidity and mortality. TB patients have been suggested to be an important population for finding of HIV infections [3], [48]. Therefore, prevention and control of co-infection is vital to reduce the epidemic of both TB and HIV/AIDS.

HIV infection has a long latent period before progression to AIDS, which makes the diagnosis, control and treatment more difficult [49]. When HIV is diagnosed early, appropriately timed interventions can lead to improved health outcomes, including slower progression and reduced mortality. Screening HIV positive patients among the high risk population has been proven to be an effective strategy in finding infections, and then later in implementing interventions [50]–[53]. As TB is one of the most common HIV-related opportunistic infections, and HIV infected persons are more susceptible to TB, several countries have recommended HIV screening for TB patients. However, targeted HIV testing, which is based on provider assessment of patient risk behaviors, fails to identify a substantial number of persons who are HIV infected. This is because many individuals may not perceive themselves to be at risk for HIV or do not disclose their risks. Therefore, routine HIV testing has been suggested to be more reasonable which also reduces the stigma associated with testing [54], [55]. Up to now, there is no policy or program implementing routine HIV testing among TB patients in mainland China. Our analyses showed that there are about 0.9% TB patients were infected with HIV, which suggested a feasibility of using routine HIV testing among TB patients to find new infections. However, the low prevalence also raises a consideration on the problem of cost-effectiveness which is an important issue should be addressed in the future studies. In addition, we observed lower summarized prevalence of HIV for population-based studies as compared to hospital-based studies. An explanation could be that TB patients who are HIV infected might be more likely to stay in the hospital because of their immune deficiency and more complicated clinical manifestation. The different prevalence of HIV between genders might be related to the potential differences of high-risk behaviors for HIV infection. However, more systematically designed studies are needed to address the factors associated with HIV infection among TB patients.

Due to the different inclusion criteria, we observed very different TB prevalence among HIV/AIDS population. For the studies performed among only AIDS patients, it was as high as 22.8%. For the studies performed among “HIV infections or AIDS patients”, however, it was only 5.2%. This is consistent with the finding that higher prevalence was observed among hospital-based studies. People with more advanced stages of AIDS are easily infected by TB or have poor capability to control disease development from latent TB infection [3], [5], [48]. We also observed significant difference between genders. However, we cannot completely exclude that such difference is attributed to potential gender-related factors, which may influence host susceptibility to the development of TB among AIDS patients. This finding might be explained by the demographic factors of the study population, such as the gender and age distribution of subjects according to disease severity, but would need verification in future studies [45]. Overall, 7.2% prevalence of TB among HIV/AIDS subjects suggests it is necessary to screen or monitor TB for this target population. In addition, the timing of Highly Active Anti-Retroviral Therapy (HAART) initiation after starting anti-TB therapy has been controversial, and optimal treatment regimens for HIV/TB co-infection are not yet clearly defined [56]. Therefore, early diagnosis of TB among HIV/AIDS cases will not only promise an earlier treatment and then better prognosis, but also provide more opportunities for deciding proper timing of HAART initiation [57].

The limitations of this study should be kept in mind. First, as positive results are more likely to be published, publication bias cannot be excluded completely, although no major publication bias was observed in the meta-analyses. Second, due to the specific high-risk behaviors for TB and HIV infection, the selection of subjects might make results prone to potential selection bias even as we have excluded two studies performed among prisoners and injecting drug users. Third, most of the included studies (27/29) were published in Chinese, quality of the reports could not be expected as well as the articles from English Journals. For example, not all necessary information, even age and gender of the study population, could be obtained from all included studies. Therefore, relevant stratified analyses (e.g. with respect to the regions or HIV infection routes) could not be performed to disclose more detailed characteristics of the co-infection and its related risk factors [45]. In addition, as prevalence of TB is very dependent on the disease stage or immune level of HIV/AIDS patients, timing of the cross-sectional survey is very important and should be considered when interpret the results. Unfortunately, very few included studies provided relevant detailed information. Finally, due to the limited number of relevant studies, incidence rate of TB and HIV co-infection was not addressed in this study.

In conclusion, our analyses suggest that it is necessary to attach importance to HIV/TB co-infection in mainland China, especially screening of TB among HIV/AIDS populations should be attached more importance which would be much helpful for treatment of both diseases. To ensure a precise estimate of the epidemic status of co-infection, further large scale or even countrywide studies are needed. Such studies will not only provide more tangible proof in promoting the development of effective strategy for diagnosis and surveillance but are also vital in reducing the prevalence of and in improving the prognosis from both TB and HIV/AIDS.

## Supporting Information

Table S1 Prevalence of HIV infection among patients with tuberculosis in mainland China (part 1/2).
(0.06 MB DOC)

Table S2 Prevalence of HIV infection among patients with tuberculosis in mainland China (part 2/2).
(0.05 MB DOC)

Table S3 Prevalence of tuberculosis among HIV/AIDS population in mainland China (part 1/2).
(0.05 MB DOC)

Table S4 Prevalence of tuberculosis among HIV/AIDS population in mainland China (part 2/2).
(0.05 MB DOC)

List S1 List of articles excluded from this study after full-text review.
(0.03 MB DOC)
