# Supplementary material for: HIV/TB Co-Infection in Mainland China: A Meta-Analysis
Source: PLoS One. 2010 May 20;5(5):e10736. doi: 10.1371/journal.pone.0010736 (PMC2873981; doi:10.1371/journal.pone.0010736)
Supplement: Table S1 — Prevalence of HIV infection among patients with tuberculosis in mainland China (part 1/2). (0.06 MB DOC) [file pone.0010736.s001.doc]

**Table S1. Prevalence of HIV infection among patients with tuberculosis in mainland China (part 1/2)**

| **First author, Published year** | **Study design** | | | | |  | **HIV screening*** | |
| --- | --- | --- | --- | --- | --- | --- | --- | --- |
|  | **Location** | **Study base**^†^ | **Duration**  **(month/year)** | **Sample size**  **n (%)** | **Mean age**  **(years)** | **Prevalence**  **n (%)** | | **Route of infection**  **n (%)** |
| Wang 2010 | GuangXi | Hospital | 12/2005-02/2006 | 2300  M: 1580 (68.7)  F: 720 (31.3) | 41.8 | 12/2300 (0.5)  M: 11/1580 (0.7)  F: 1/720 (0.1) | | NA |
| Li 2009 | BeiJing | Population | 10/2007 | 476 | NA | 1/476 (0.2) | | IDU: 1 |
| Tang 2009 | GuangXi | Hospital | 10-12/2007 | 341 | NA | 8/341 (2.3) | | NA |
| Cao 2008 | GuangXi | Hospital | 2001-2005 | 6573  M: 4212 (64.1)  F: 2361 (35.9) | NA | 86/6573 (1.3)  M: 64/4212 (1.5)  F: 22/2361 (0.9) | | IDU: 36 (41.9)  Sex: 27 (31.4)  IDU and Sex: 10 (11.6)  Blood: 2 (2.3)  Uncertain: 11 (12.8) |
| Li 2008 | ShanDong | Population | 09-12/2007 | 1792  M: 1292 (72.1)  F: 500 (27.9) | NA | 2/1792 (0.1)  M: 2/1292 (0.2)  F: 0/500 (0) | | Blood: 2 (100) |
| Lu 2008 | HeBei | Population | 01-09/2007 | 844 | NA | 0/844 (0) | | NA |
| Peng 2008 | GuangXi | Population | 01/2007-03/2008 | 533 | NA | 13/533 (2.4) | | NA |
| Shi 2008 | HeBei | Hospital | 2004-2006 | 412 | NA | 2/412 (0.5) | | NA |
| Wang 2007 | GuangXi | Hospital | 05-08/2005 | 580 | NA | 16/580 (2.8) | | NA |
| He 2006 | GuangXi | Hospital | 01/2004-12/2005 | 2463  M: 1946 (79.0)  F: 517 (21.0) | M: 39  F: 33 | 106/2463 (4.3)  M: 93/1946 (4.8)  F: 13/517 (2.5) | | IDU: 50 (47.2)  Sex: 26 (24.5)  Blood: 3 (2.8)  Uncertain: 27 (25.5) |
| Lin 2006 | GuangXi | Population | 09-10/2005 | 3698  M: 2473 (66.9)  F: 1225 (33.1) | NA | 50/3698 (1.4)  M: 43/2473 (1.7)  F: 7/1225 (0.6) | | Blood: 4 (8.0)  IDU: 30 (60.0)  Uncertain: 16 (32.0) |

Abbreviation: F, female; HIV, human immunodeficiency virus; IDU, injecting drug user; M, male; NA, not available.

* Please refer Methods and Materials with respect to study base and diagnosis of HIV infection.
