# Supplementary material for: HIV/TB Co-Infection in Mainland China: A Meta-Analysis
Source: PLoS One. 2010 May 20;5(5):e10736. doi: 10.1371/journal.pone.0010736 (PMC2873981; doi:10.1371/journal.pone.0010736)
Supplement: Table S2 — Prevalence of HIV infection among patients with tuberculosis in mainland China (part 2/2). (0.05 MB DOC) [file pone.0010736.s002.doc]

**Table S2. Prevalence of HIV infection among patients with tuberculosis in mainland China (part 2/2)**

| **First author, Published year** | **Study design** | | | | |  | **HIV screening*** | |
| --- | --- | --- | --- | --- | --- | --- | --- | --- |
|  | **Location** | **Study base*** | **Duration**  **(month/year)** | **Sample size**  **n (%)** | **Mean age**  **(years)** | **Prevalence**  **n (%)** | | **Route of infection**  **n (%)** |
| Jin 2005 | GuangXi | Hospital | 1998-2003 | 9887 | NA | 129/9887 (1.3) | | NA |
| Wang 2004 | GuangDong | Hospital | 08/2002-07/2003 | 326 | NA | 0/326 (0) | | NA |
| Zhen 2004 | HeNan | Hospital | NA | 1569  M: 1081 (68.9)  F: 488 (31.1) | 41 | 15/1569 (1.0)  M: 13/1081 (1.2)  F: 2/488 (0.4) | | Blood: 2 (13.3)  Uncertain: 13 (86.7) |
| Wang 2003 | GuangDong | Hospital | 09/2001-12/2002 | 376  M: 297 (79.0)  F: 79 (21.0) | NA | 1/376 (0.3) | | NA |
| Zhou 2002 | XinJiang | Hospital | 1998-2000 | 200  M: 123 (61.5)  F: 77 (38.5) | NA | 9/200 (4.5)  M: 8/123 (6.5)  F: 1/77 (1.3) | | NA |
| Feng 2001 | GuangDong | Hospital | 03/1998-06/1999 | 2151  M: 1565 (72.8)  F: 586 (27.2) | NA | 7/2151 (0.3)  M: 5/1565 (0.3)  F: 2/586 (0.3) | | Blood: 3 (42.9)  Sex: 4 (57.1) |
| Li 2001 | HeNan | Hospital | 03-12/2000 | 607 | NA | 2/607 (0.3) | | NA |
| Kong 1999 | BeiJing | Hospital | 02/1995-05/1998 | 2973  M: 2153 (72.4)  F: 820 (27.6) | NA | 3/2973 (0.1)  M: 2/2153 (0.1)  F: 1/820 (0.0) | | IDU: 1 (33.3)  Sex: 2 (66.7) |

Abbreviation: F, female; HIV, human immunodeficiency virus; IDU, injecting drug user; M, male; NA, not available.

* Please refer Methods and Materials with respect to study base and diagnosis of HIV infection.
