# Supplementary material for: HIV/TB Co-Infection in Mainland China: A Meta-Analysis
Source: PLoS One. 2010 May 20;5(5):e10736. doi: 10.1371/journal.pone.0010736 (PMC2873981; doi:10.1371/journal.pone.0010736)
Supplement: Table S3 — Prevalence of tuberculosis among HIV/AIDS population in mainland China (part 1/2). (0.05 MB DOC) [file pone.0010736.s003.doc]

**Table S3. Prevalence of tuberculosis among HIV/AIDS population in mainland China (part 1/2)**

| **First author, Published year** | **Study design** | | | | | |  | **Characteristics of TB patients*** | |
| --- | --- | --- | --- | --- | --- | --- | --- | --- | --- |
|  | **Location** | **Study base*** | **Enrollment duration**  **(month/year)** | **Sample size**  **n (%)** | **Age**  **mean/range**  **(years)** | **Route of infection**  **n (%)** | **Prevalence**  **n (%)** | | **Microbiologically confirmed cases**  **n/N (%)** |
| Li 2009 | BeiJing | Population  (HIV/AIDS) | 10/2007 | 152 | NA | NA | 14 (9.2) | | NA |
| Wei 2009 | GuangXi | Hospital  (AIDS) | 08/2007-12/2008 | 386  M: 215 (55.7)  F: 171 (44.3) | 34 (23-69) | NA | 64 (16.6) | | NA |
| Yu 2009 | GuangXi | Hospital  (HIV/AIDS) | 08/2006-03/2007 | 660  M: 449 (68.0)  F: 211 (32.0) |  | NA | 151 (22.9)^&^  M: 116 (25.8)  F: 35 (16.6) | | 56/151 (37.1)^†^ |
| Qian 2009 | ShanXi | Hospital  (HIV/AIDS) | 2007 | 194  M: 119 (61.3)  F: 75 (38.7) | NA | Blood: 194 | 9 (4.6)  M: 5 (4.2)  F: 4 (5.3) | | 2/9 (22.2) |
| Tang 2009 | GuangXi | Hospital (AIDS) | 10-12/2007 | 778 | NA | NA | 151 (19.4) | | NA |
| Lu 2008 | HeBei | Population  (HIV/AIDS) | 03-10/2007 | 397  M: 165 (41.6)  F: 232 (58.4) | 20-59^#^ | Sex: 46 (11.6)  Blood: 307 (77.2)  Mother to child: 26 (6.6)  Uncertain: 18 (4.6) | 10 (2.5)  M: 4 (2.4)  F: 6 (2.7) | | 3/10 (30.0) |
| Peng 2008 | GuangXi | Population  (HIV/AIDS) | 01/2007-03/2008 | 192 | NA | NA | 17 (8.9) | | NA |
| Shi 2008 | HeBei | Hospital (HIV) | 04/2004-12/ 2006 | 53  M: 27 (50.9)  F: 26 (49.1) | NA | NA | 4 (7.5) | | NA |

Abbreviation: AIDS, acquired immune deficiency syndrome; F, female; HIV, human immunodeficiency virus; IDU, injecting drug user; M, male; NA, not available; TB, tuberculosis.

* Please refer Methods and Materials with respect to study base and diagnosis of TB.

^&^ CD4 cell count was less than 200/mL for 88% cases.

^†^ Differential identification of Mycobacteria in sputum cultures showed a 38.5% infection of Mycobacterium other than TB.

^#^ Most of the subjects (92%) were aged in 20-59 years.
