# Supplementary material for: HIV/TB Co-Infection in Mainland China: A Meta-Analysis
Source: PLoS One. 2010 May 20;5(5):e10736. doi: 10.1371/journal.pone.0010736 (PMC2873981; doi:10.1371/journal.pone.0010736)
Supplement: Table S4 — Prevalence of tuberculosis among HIV/AIDS population in mainland China (part 2/2). (0.05 MB DOC) [file pone.0010736.s004.doc]

**Table S4. Prevalence of tuberculosis among HIV/AIDS population in mainland China (part 2/2)**

| **First author, Published year** | **Study design** | | | | | |  | **Characteristics of TB patients*** | |
| --- | --- | --- | --- | --- | --- | --- | --- | --- | --- |
|  | **Location** | **Study base*** | **Enrollment duration**  **(month/year)** | **Sample size**  **n (%)** | **Age**  **mean/range**  **(years)** | **Route of infection**  **n (%)** | **Prevalence**  **n (%)** | | **Microbiologically confirmed cases**  **n/N (%)** |
| Wang 2008 | AnHui | Population  (HIV/AIDS) | 04/2005-08/2006 | 150 | 42.9 | Blood: 138 (92.0)  Mother to child: 2 (1.3)  Uncertain: 2 (1.3) | 5 (3.3) | | 0/5 |
| Wang 2007 | GuangXi | Hospital (AIDS) | 03-08/2005 | 260 | NA | NA | 91 (35.0) | | NA |
| Yin 2007 | HuBei | Population  (HIV/AIDS) | 01-03/2005 | 179  M: 107 (59.8)  F: 72 (40.2) | NA | NA | 12 (6.7)^&^  M: 10 (9.3)  F: 2 (2.8) | | 10/12 (83.3) |
| Cao 2006 | HeNan | Population  (HIV/AIDS) | 06-08/2005 | 5873  M: 2772 (47.2)  F: 3101 (52.8) | 11-85 | NA | 28 (0.5)  M: 22 (0.8)  F: 6 (0.2) | | 13/28 (46.4) |
| Liu 2006a | HuBei | Population  (HIV/AIDS) | 2005 | 140  M: 96 (68.6)  F: 44 (31.4) | NA | Sex: 13 (9.3)  Blood: 92 (65.7)  IDU: 1 (0.7)  Uncertain: 35 (25.0) | 3 (2.1)  M: 2 (2.0)  F: 1 (2.3) | | 2/3 (66.7) |
| Liu 2006b | GuangXi | Population  (HIV/AIDS) | 06/2006 | 2341 | NA | NA | 149 (6.4) | | 10/149 (6.7) |
| Zhou 2006 | ShanXi | Population (HIV/AIDS) | 06-07/2006 | 390  M: 217 (55.6)  F: 173 (44.4) | 4-72 | Sex: 48 (12.3)  Blood: 315 (80.8)  Mother to child: 16 (4.0)  Uncertain: 13 (3.3) | 36 (9.2)  M: 23 (10.6)  F: 13 (7.5) | | 21/36 (58.3) |

Abbreviation: AIDS, acquired immune deficiency syndrome; F, female; HIV, human immunodeficiency virus; IDU, injecting drug user; M, male; NA, not available; TB, tuberculosis.

* Please refer Methods and Materials with respect to study base and diagnosis of TB.

^&^ CD4 cell count was less than 200/mL for 25% cases.
