# Supplementary material for: HIV/TB Co-Infection in Mainland China: A Meta-Analysis
Source: PLoS One. 2010 May 20;5(5):e10736. doi: 10.1371/journal.pone.0010736 (PMC2873981; doi:10.1371/journal.pone.0010736)
Supplement: List S1 — List of articles excluded from this study after full-text review. (0.03 MB DOC) [file pone.0010736.s005.doc]

**List S1. List of articles excluded from this study after full-text review**

***2 addressing specific high risk populations:***

1. Li Y, Li H, Fu J. HIV test among 382 drug users who are affacted by TB Chinese Journal of AIDS & STD 2006;12:405.
2. Yang P, Ai B, Liu H, et al. HIV and TB co-infection among prisoners. Chinese Journal of Public Health 2006;22:535-6.

***4 based on serological TB diagnosis only:***

1. Zheng S, DuanMU H, Li Z, et al. Study of association between immune levels and tuberculin test responses among cases with HIV infection. The Journal of the Chinese Antituberculosis Association 2002;24:320-3.
2. Wei X, Xu Y, Xue M, Ai W, Zhang J. Co-infection of hepatitis B virus, hepatitis C virus and syphilis among cases infected with human immunodeficiency virus. JiangXi Journal of Medical Laboratory Sciences 2006;24:283-4.
3. Wang H, Tan Y, Zhu XY, et al. Use of IFN-γ release assay in latent tuberculosis infection diagnosis in general and HIV-infected populations. Chinese Journal of Microbiology and Immunology 2009; 29:1037-41.
4. Qi MC, Li SL. TB infection status among 156 HIV infections. Laboratory Medicine and Clinic 2009; 11:1977.

***3 from regions of China other than mainland:***

1. Wong K, Lee S, Lo Y, et al. Profile of opportunistic infections among HIV-1 infected people in Hong Kong. Zhonghua Yi Xue Za Zhi (Taipei) 1995;55:127-36.
2. Chan KC, Tang HW, Wong K. Prevalence of purified protein derivative positivity in human immunodeficiency virus infected individuals in Hong Kong. Chin Med J (Engl) 2002;115:1091-2.
3. Chiang CY, Wu IH, Yu MC, et al. Screening of human immunodeficiency virus infection in pulmonary tuberculosis patients in Taiwan. J Formos Med Assoc 1998;97:66-8.

***3 on incidence rates:***

1. Shi P, Wang J, Liu X, Huang Q, Sun H, Lu H. Clinical report of 31 HIV/TB co-infections. Journal of Clinical Internal Medicine 2007;24:278.
2. Li S, Zhao D, Liu G, et al. Research and analysis of the status of acquired immunodeficiency syndrome complicated with tuberculosis-----observation of 18 clinical cases. HeNan Journal of Prevention Medicine 2008;19
3. Ayimuxia Y, Yan X. Analysis of 80 HIV/TB co-infected cases. Chinese Community Doctors 2006;22:28.

***5 published in duplication:***

1. Zeng C, Feng D, Lin P, et al. Correlation analysis between HIV/AIDS and TB. Chinese Journal of Std & Aids Prevention and Control 2001;7:5-6.
2. Lu L, Zhang L, Li X, Zhang P, Zhao S, Zhang X. Analysis on Coincidence of HIV／AIDS and Tuberculosis in 4 Counties of Hebei Province. Practical Preventive Medicine 2008;15:106-7.
3. Zhang P, Zhang Z, Dong X. Analysis on the Screening Results of Tuberculosis in Patients with HIV／AIDS. Journal of Practiceal Medical Techniques 2007;14:5034-5.
4. Qu S, Lu L, Sun Y, Qu Y, Zhang X, Xu Q. Investigation of TB co-infection among HIV/AIDS patients. Journal of Practiceal Medical Techniques 2006;5:2714.
5. Jiang X, Lu H, Zhang Y, et al. A cross-sectional study of HIV and tuberculosis coinfection cases in mainland China. South Med J 2008;101:914-7.

***6 with a sample size < 50:***

1. Cui W, Wang L, Yang Y, et al. Research on prevention and treatment against tuberculosis in HIV/AIDS patients. HeNan Journal of Prevention Medicine 2003;14:262-4.
2. Yang C, Tang F. Clinical analysis of 32 cases with HIV/TB co-infection. Chinese Journal of AIDS & STD 2007;13:385.
3. Maimaitiaili A, Song J, Zuohela T, Adili., Pan K, Zhang Y. Analysis on clinical characteristics 0f 40 patientions with AIDS in XinJiang. Chinese Journal of Infection Control 2008;7:389-91.
4. Liu G, Cui W, Wang L, et al. Research on HIV/MTB infected patients. Chinese Journal of STD & AIDS Prevention and Control 2002;8:82-3.
5. Sun H. Analyaia of 7 cases with HIV/TB co-infection. SiChuan Medical Journal 2004;25:1141.
6. Xu X, Li X. Clinical analysis of 20 cases with HIV/TB co-infection. Journal of Clinical Pulmonary Medicine 2006;11:231.
